# Resident attendance at weekly conferences after implementation of an optional asynchronous learning curriculum

**DOI:** 10.5116/ijme.58fc.872c

**Published:** 2017-05-11

**Authors:** Abbas Kothari, Alan H. Breaud, A. Travis Manasco, Jordan A. Spector, Jolion McGreevy, Alexander Y. Sheng

**Affiliations:** 1 Department of Emergency Medicine, Boston Medical Center, USA

**Keywords:** Asynchronous learning, curriculum design, residency administration, conference attendance, educational planning

## Introduction

Asynchronous learning (ASL) is a student-centered method of teaching utilizing online learning resources to overcome time and space constraints to facilitate information sharing and interaction amongst learners.^1^ Studies show it to be a valued and effective method of learning.^2^ As the availability of online educational resources for physicians has increased in recent years,^3^ Free Open Access Medical Education (FOAM) has become a familiar term to many. Some have suggested that ASL via FOAM can be a more flexible and cost-effective teaching method compared to conventional methods.^4^ In fact, FOAM may be preferred by the current generation of ‘millennial’ learners.^5^^,^^6^

Despite its increasing prevalence and utilization, FOAM is not a panacea. Educators who support a constructivist epistemology for education argue that the inherent isolation of ASL (studying alone at a private computer workstation) is a detriment. In addition, ASL can decrease learner satisfaction by diminishing a student’s sense of connection to peer learners.^7^  As such, blended learning, a hybrid combining classroom and online learning, creates a stronger sense of community amongst learners than either traditional or online courses alone.^8^

The Emergency Medicine (EM) Residency Review Committee (RRC) encourages programs to supplement the weekly educational conference curriculum with ASL in the form of “Individualized Interactive Instruction (III)”.^9^ In response, our EM residency program devised and implemented an optional, Web-based, asynchronous learning curriculum (ASYNC) to supplement our traditional planned weekly residency educational conferences, in essence creating an optional ”blended curriculum”. Consistent with RRC recommendations, we permitted residents to utilize ASYNC to obtain up to 20% of required residency educational conference attendance as extra credit (the equivalent of one hour out of the five offered each week within the extant didactic curriculum).  The American College of Graduate Medical Education (ACGME)-mandated 70% conference attendance rate served as an inherent incentive to participate in ASYNC.^9^

There is no existing evidence to inform whether introducing an asynchronous learning curriculum will have unintended consequences such as lowering attendance to planned educational activities. Considering the current generation of ‘millennial’ learners’ preference for FOAM,^5^ it is conceivable that implementation of ASYNC would be associated with a reduction in attendance rates within the EM didactic curriculum. Such experiences may give educators and program directors pause before implementing an analogous curriculum.

### ASYNC Design and Implementation

We implemented ASYNC in February 2015 at an urban, academic medical center which hosts a postgraduate year (PGY) 1-4 EM training program of 48 residents. ASYNC consists of two components, devised and supervised by residency program directors. The first portion incorporates Academic Life in Emergency Medicine’s (ALiEM) Approved Instructional Resources (AIR) series.^10^ Since most of the curated FOAM content from AIR comprises of high-yield evidence-based summaries of medical literature, we developed the second part of ASYNC to encourage the primary appraisal of the literature to promote self-directed assessment seeking and reflection. This second component of ASYNC consists of two monthly high-impact journal articles, selected by faculty. Learners are expected to read the articles and contribute to an online discussion board designed to encourage interaction amongst residents and faculty regarding if and how the literature can be translated into clinical practice.

### Participation in ASYNC

Residents receive a single email to encourage participation each month.  The email contains links to both the assigned AIR module and the two articles up for review and discussion.  Participation in AIR was defined as completion of post-module quizzes provided by ALiEM. Participation in high-impact journal article online discussions was defined as having answered four open-ended questions about the strengths, limitations, outcomes, and implications of the article on the online discussion board. More than half of the residents participated in ASYNC to some degree during its first year of implementation. Several learners participated every month, though a larger portion of residents took part on a more sporadic basis. Since ASYNC was an optional component of the curriculum and we were uncertain of its potential impact on resident attendance to planned weekly educational conferences, no additional efforts were made to promote participation in ASYNC.

### Resident Attendance Before and After

The average residency educational conference attendance across all PGY-levels remained unchanged the year after (Feb 2015-Jan 2106) ASYNC’s implementation as compared to the year before (Feb 2014-Jan 2015). 

Due in part to the demanding clinical schedules and the ACGME stipulated work-hour regulations; it is nearly impossible for EM residents to attend 100% of the planned residency educational conferences during their training. As a result, residency program directors and educational planners have to make critical decisions regarding curriculum content and format with time and space constraints in mind. The use of FOAM/III as ASL can provide valuable learning opportunities to supplement residency educational conferences.

We are the first to examine a potential unintended consequence of implementing an ASL curriculum. If doing so significantly impacts attendance at weekly planned didactics, residency program leadership will need to reconsider the resources, time, and effort put into the extant classroom program. Such experiences might necessitate a major curricular redesign. In our residency program, resident attendance to weekly residency educational conference did not change after the implementation of the ASYNC. With consideration of the social learning framework within residency didactics, for those who perceive constructivism as key to learning and knowledge acquisition during residency, our findings may be reassuring.  

## Conclusions

Resident attendance at the weekly residency educational conference did not change after the implementation of the ASYNC within our residency program. Our experience should provide some reassurance to educators considering the implementation of an analogous curriculum.

Nevertheless, our experience is limited to a single institution for only one year after our intervention. We will continue to monitor residency attendance to weekly educational conference going forward. If the non-negative trend in attendance continues, and our experience is replicated at other centers tracking attendance for longer observation periods, we would consider incorporating ASYNC as part of our formal required curriculum and potentially replacing a portion of in-person didactics with ASYNC. Doing so would be supported by educational literature demonstrating asynchronous learning curriculums to be a valued and effective method of learning preferred by the current generation of ‘millennial’ learners.^2^^,^^5^^,^^6^ 

### Acknowledgements

The authors would like to humbly acknowledge Drs. Michelle Lin, Andy Grock, and the entire team at Academic Life in Emergency Medicine for making the innovative and impactful Approved Instructional Resources series available to the entire emergency medicine community. We would also like to thank our residency coordinator Nancy Connor for meticulously tracking our residency educational conference attendance over the years.

### Conflict of Interest

The authors declare that they have no conflict of interest.
